# Agglomeration structure of superparamagnetic nanoparticles in a nematic liquid crystal medium: Image analysis datasets based on cryo-electron microscopy and polarised optical microscopy techniques

**DOI:** 10.1016/j.dib.2021.106716

**Published:** 2021-01-11

**Authors:** Baeckkyoung Sung, Leon Abelmann

**Affiliations:** a KIST Europe Forschungsgesellschaft mbH, 66123 Saarbrücken, Germany; b Liquid Crystal Institute and Chemical Physics Interdisciplinary Program, Kent State University, Kent, OH 44242, United States; c Division of Energy & Environment Technology, University of Science & Technology, 34113 Daejeon, Republic of Korea; d MESA+ Institute for Nanotechnology, University of Twente, 7500 AE Enschede, the Netherlands

**Keywords:** Iron oxide nanoparticle, Aggregation, Liquid crystal, Topological defect, Cryogenic transmission electron microscopy (cryo-TEM), Polarised light microscopy

## Abstract

This dataset shows the agglomerate dimension and structure of oleic acid-coated superparamagnetic nanoparticles (SPIONs), which are dispersed in the nematic fluid of a thermotropic liquid crystal (LC), 4-cyano-4′-pentylbiphenyl (5CB). The analysed datasets were acquired from the raw images of the SPION-5CB mixtures obtained using cryogenic transmission electron microscopy (cryo-TEM) and polarised optical microscopy. The image data were quantitatively analysed to extract statistical information on the sizes of SPIONs and their agglomerates and the inter-particle spacing of the agglomerated SPIONs. This dataset supports the fundamental understanding on how colloidal nanospheres behave in an anisotropic fluid, and has a potential to be used as a part of database for automated design of new hybrid materials.

## Specifications Table

**Table:** 

Subject	Liquid crystals, magnetic nanoparticles
Specific subject area	Soft matter, liquid crystal physics, colloid physics and chemistry, transmission electron microscopy, optical imaging
Type of data	Graph
How data were acquired	Cryo-TEM, polarised light microscopy, image analysis software (ImageJ and Fiji)
Data format	Raw and Analysed
Parameters for data collection	Cryo-TEM: vitrified LC in a perforated carbon film (thickness = 12 nm, hole diameter = 2 µm) for TEM operation at < −170 °C Polarised light microscopy: LC cell with the distance between the two glass surfaces = 10.5 ± 0.8 µm, for observation at 22–25 °C (nematic phase)
Description of data collection	The raw data were collected from oleic acid-coated SPION-doped nematic 5CB fluid under confinement of a thin glass cell, prepared for polarised light microscopy observation at room temperature. For cryo-TEM, the fluid was rapidly vitrified under the confinement of a micro-perforated carbon film-coated grid, transferred to a cryo-holder, and then imaged with low electron dose. The images were processed and analysed with ImageJ or Fiji software.
Data source location	Institution: Liquid Crystal Institute City/Town/Region: Kent, OH Country: USA Latitude and longitude for collected samples/data: N 41° 8′ 39, W 81° 20′ 24
Data accessibility	4TU Repository: https://doi.org/10.4121/13365359.v1
Related research article	B. Sung, H. Yan, C. Kim, L. Abelmann, Inhomogeneous nematic-isotropic phase transition of a thermotropic liquid crystal doped with iron oxide nanoparticles, Phys. Lett. A 384 (2020) 126,927. https://doi.org/10.1016/j.physleta.2020.126927

## Value of the Data

• This dataset contains cryo-TEM snapshots, together with the polarised optical microscopy images, visualising the behaviour of nanoparticles suspended in a host liquid crystal, which is one of the fundamental questions in the soft matter field.
• These data are useful for the theoretical and experimental study of *in situ* morphology and structure of nanosphere agglomerates in elastic and anisotropic fluids, as well as the engineering of materials and devices that combine the properties of liquid crystals and magnetic nanoparticles.
• These data can provide reference values for the development of nano-hybrid optical and magnetic devices, and can be used as a database source for the computational design of novel functional materials.

## 1 Data Description

**Fig. 1:**
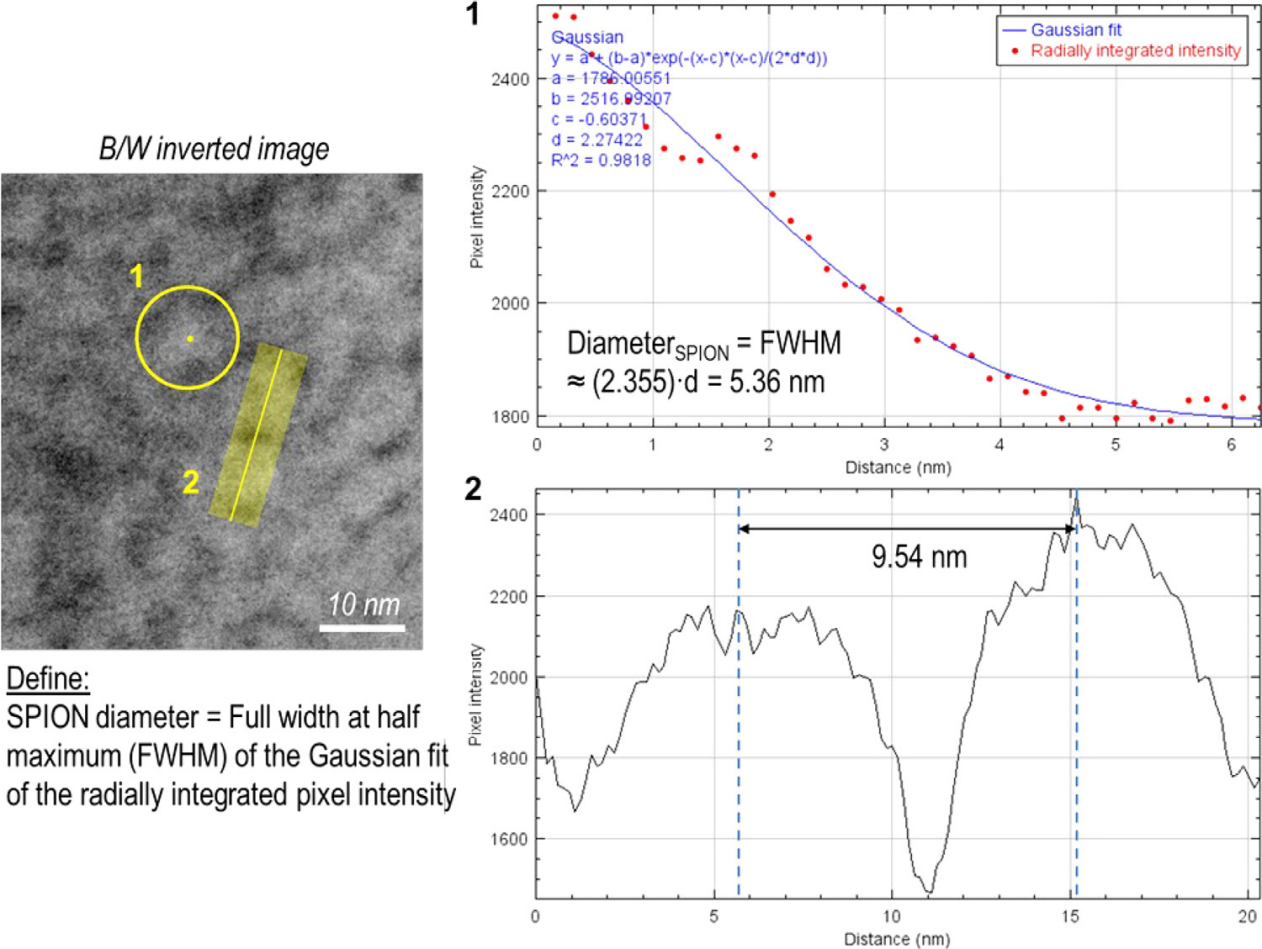
Image analysis method for measuring the oleic acid-coated SPION diameter and inter-SPION distance inside the agglomerates, suspended in the 5CB matrix. Raw data values are available in the 4TU repository.

For the measurements of the oleic acid-coated SPION diameter and inter-SPION spacing, we used the radial integration profile and line profile modthds, respectively, using ImageJ (or Fiji) software and its plug-in modules (Fig. 1). First, the cryo-TEM images have been black/white inverted so that the SPION part exhibits higher grayscale levels than the background. (1) To measure the SPION diameter, the pixel intensity for each SPION (+ surrounding region) was radially integrated at full angle, and the profile was fitted to a Gaussian, from which the standard deviation (d) value could be obtained. Then, the full width at half maximum (FWHM) of the Gaussian function was simply calculated from the relation, FWHM ≈ 2.355d, which determined the SPION diameter. (2) To measure the inter-SPION spacing, the width-controlled line profile plot was applied to cover the entire region of 2 adjacent SPIONs. Then, the distance between the local maxima at the centres of both peaks was measured to be the centre-to-centre spacing between the SPIONs.

**Fig. 2:**
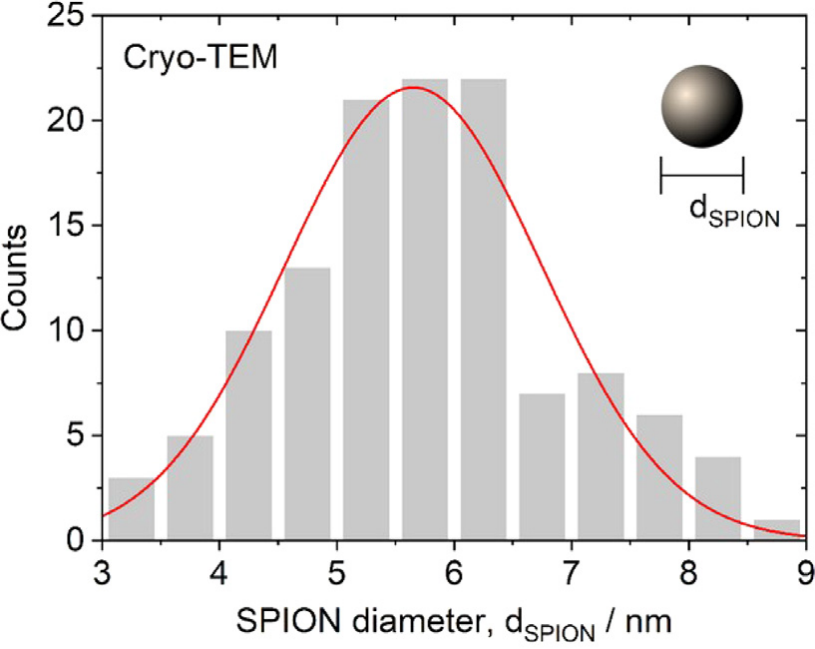
Distribution of SPION diameter, d_SPION_, measured by cryo-TEM (*n* = 122). Raw data values are available in the Repository.

**Fig. 3:**
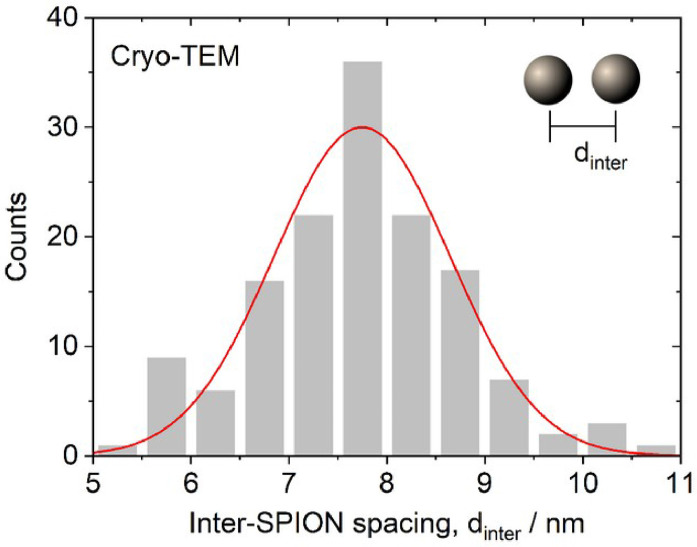
Distribution of inter-SPION spacing, d_inter_, within the agglomerations in the 5CB matrix, measured by cryo-TEM (*n* = 142). Raw data values are available in the Repository.

**Fig. 4:**
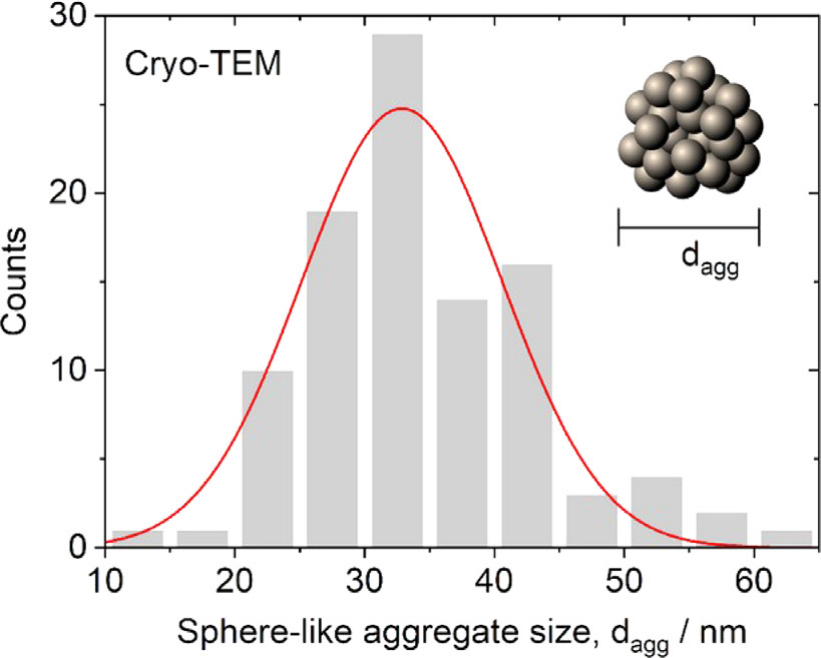
Distribution of the sphere-like aggregate size, d_agg_, in 5CB matrix measured by cryo-TEM (*n* = 100). Raw data values are available in the Repository.

**Fig. 5:**
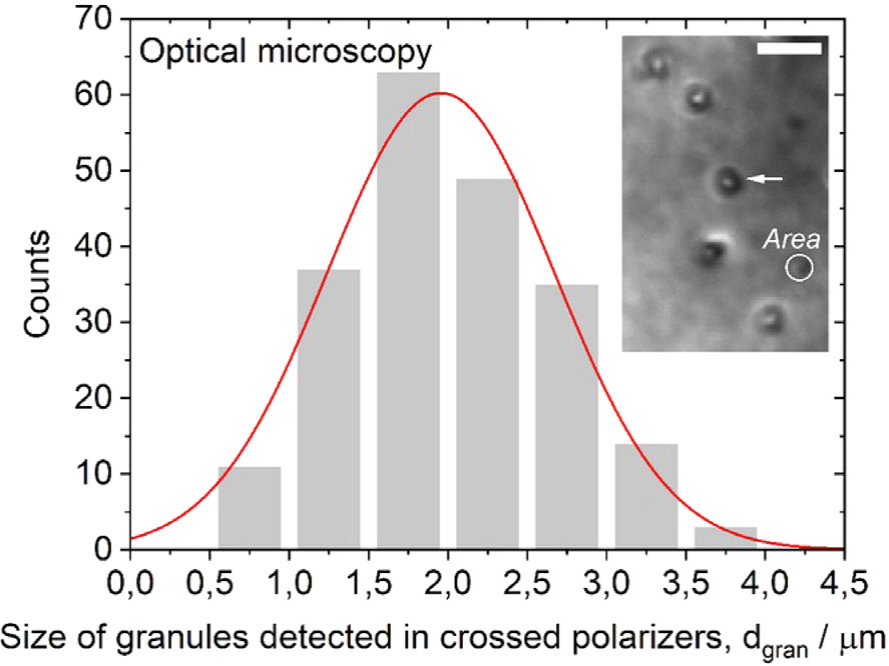
Distribution of the size of granules, d_gran_, in the 5CB matrix measured by polarised optical microscopy (*n* = 212). Scale bar, 10 µm. Raw data values are available in the Repository.

In the cryo-TEM images, the agglomerates predominantly exhibited clustered sphere-like aggregates of SPIONs [1]. The mean diameter of SPIONs, which were synthesised according to the method by Yan et al. [2], was measured to be 5.8 ± 0.1 nm and the distribution had a standard deviation of 1.2 nm (Fig. 2). This distribution was close to normal; the optimal fit to a Gaussian distribution was centred at 5.7 nm with a standard deviation of 1.1 nm. For the inter-SPION spacing inside the agglomerates, the average spacing was 7.72±0.08 nm and the distribution had a standard deviation of 1.0 nm (Fig. 3). The fitted curve showed a Gaussian distribution centred at 7.75 nm with a standard deviation of 0.9 nm. The average size of the sphere-like aggregates was 34.7 ± 0.9 nm, the distribution had a standard deviation of 9 nm (Fig. 4). The fitted curve showed a Gaussian distribution centred at 32.9 nm with a standard deviation of 8 nm. In the polarised optical microscopy images, the agglomerates appeared as granular structures (Fig. 5). As shown in the inset of Fig. 5, for the measurement of d_gran_, the granule's area, A, was converted to the granule size according to the relation, $d_{gran}=2\sqrt{A/\pi }$. The average granule size was 2.03±0.05 µm, with a standard deviation of 0.7 µm. The fitted curve showed a Gaussian distribution centred at 1.96 µm with a mean standard deviation of 0.7.

## 2 Experimental Design, Materials and Methods

All image analyses and measurements were performed using Fiji/ImageJ, which is a Java-based software developed and distributed by the National Institute of Health (NIH; Bethesda, MD, USA) and the Laboratory for Optical and Computational Instrumentation (University of Wisconsin, Madison, WI, USA). The Fiji, an image processing package based on ImageJ, was downloaded from the website of NIH (https://imagej.nih.gov/ij/docs/guide/146–2.html). Additionally, the *Radial Profile Extended Plugin* was downloaded (http://questpharma.u-strasbg.fr/html/radial-profile-ext.html). The cryo-TEM method was conducted in accordance to the previously reported methods [3], [4].

In the Fiji software, the cryo-TEM images were opened and black/white inverted, then the SPION diameter was measured by performing: “Plugins” → “Radial Profile Angle” → Select ROI (X-Y centres & radius): Set full angle integration → “Calculate Radial Profile” → “List” → “Edit” → “Select All”. Finally, the result was right-clicked and plotted by selecting “Plot”. In the new window, the plot was curve-fitted by performing: “analyse” → “Tools” → “Curve Fitting” → Copy the plot values and paste in the text field of *Curve Fitter* window → Select “Gaussian” in the *Curve Fitter* → “Fit”.

In the Fiji software, the inter-SPION spacing measurement was done using the *Line Selection Tools* (straight line) through: (i) drawing a line across the centres of two adjacent SPIONs, (ii) double-clinking the *Line Selection Tools* icon to generate the *Line Width* controller, (iii) adjusting the line width to fully cover the SPION areas, (iv) selecting “analyse” → “Plot Profile”, and (v) measuring the x-coordinates of the local maxima at the centres of both intensity profile peaks.

The polarised light microscopy was conducted in accordance to the method reported in Sung et al. [1]. For the size measurement of the granules, using the Fiji software, ellipsoid was drawn (“Oval” icon) to fit cover a single granule on the opened image, and the ellipsoid area was obtained by choosing “analyse” → “Measure”, then the area was directly converted to the diameter value. All the data in spreadsheets were plotted, fitted, and statistically analysed using the software OriginPro 2019b (OriginLab, Northampton, MA, USA).

## Declaration of Competing Interest

The authors declare that they have no known competing financial interests or personal relationships which have, or could be perceived to have, influenced the work reported in this article.
